# Should acupuncture therapy be used for acute facial paralysis? A protocol for systematic review

**DOI:** 10.1186/s13643-023-02194-5

**Published:** 2023-03-15

**Authors:** Lu Cheng, Xiao-lin Li, Yi Ying, Shi-hao Du, Xu-dong Zhang, Wei Guo, Shu-qi Mi, Ji-ping Zhao

**Affiliations:** 1 Chinese PLA General Hospital, No. 28 Fuxing Road, Haidian District, Beijing, China; 2 Dongfang Hospital Beijing University of Chinese Medicine, No. 6 Fangxingyuan, Fengtai District, Beijing, China; 3 Department of Pharmacology, School of Basic Medical Sciences, Peking University, Beijing, China; 4 Institute of Acupuncture and Moxibustion, China Academy of Chinese Medical Sciences, Beijing, China; 5 Department of Traditional Chinese Medicine, Beijing Jishuitan Hospital, Xicheng District, Beijing, China; 6 Beijing University of Chinese Medicine Affiliated Dongzhimen Hospital, No. 5 Haiyuncang, Dongcheng District, Beijing, China; 7 College of Acupuncture-Moxibustion and Tuina, Hebei University of Chinese Medicine, No. 3 Xingyuan Road, Luquan District, Shijiazhuang, Hebei Province China

**Keywords:** Acupuncture therapy, Peripheral facial paralysis, Acute phase, Systematic review, Protocol

## Abstract

**Background:**

Peripheral facial paralysis (PFP) results in functional disorder and social dysfunction, when it is under a severe condition at onset, long-term poor outcomes do occur. Different acupuncture methods have been reported to be potentially effective for shortening the disease course and reducing the occurrence of sequelae when they are applied at an early stage. Neuro edema is a common pathological feature in the acute phase, and many clinical studies have suggested its effect of reducing facial nerve edema. It is of value to estimate the effectiveness and safety of acupuncture treatment at the onset, and to assess the most suitable acupuncture method for the acute period.

**Methods and analysis:**

All the RCTs and quasi-RCTs on acupuncture therapy for patients who is during acute stage of PFP will be included. The recovery rate of facial function, the time it takes to restore facial function and the odds of sequelae occurring will be the key parts we focus on. Psychological well-being and quality of life will also be evaluated. Literature searching will be conducted until December 31th, 2022 from eight databases systematically. Two reviewers will screen the literature and extract the data independently. RevMan software will be used for data analysis, and the version 2 of the Cochrane risk-of-bias tool (RoB 2) will be used to assess the certainty of evidence. Forest plots and summary findings will be generated. If data permits, a meta-analysis will be conducted.

**Ethics and dissemination:**

Since this study will not involve clinical treatment of patients, ethics approval is not required. The result of this study will be submitted to a peer-reviewed journal for publication and as a proposal for clinical practice and further study on acupuncture treatment at the early stage of PFP.

**Discussion:**

This review will summarize the evidence on the different type of acupuncture therapy for acute Bell’s palsy and Ramsay-Hunt syndrome. We anticipate that it would be safe and effective when applied to the acute phase of PFP, and some specific suitable acupuncture methods would be found resulting from this review.

**Systematic review registration:**

International Prospective Register for Systematic Reviews (PROSPERO) number CRD42020205127

**Supplementary Information:**

The online version contains supplementary material available at 10.1186/s13643-023-02194-5.

## Background

Facial paralysis (FP) results in functional disorder and social dysfunction, which is related to the inability to control the muscles of facial expression [1–3]. In all types of facial paralysis, Bell’s palsy is the most common acute disorder affecting a single nerve, yet the cause of the disease is unclear. This condition affects 11–40 people per 100,000 in the population each year, most commonly among the age group of 30–45 years old [4]. Compared with Bell’s palsy, facial palsy caused by Varicella Zoster Virus (VZV), which is best known as the Ramsay Hunt syndrome has a lower incidence but severer symptoms at onset, and it is less likely to be cured [5]. Besides, a common complication of Ramsay Hunt syndrome which is a chronic pain condition known as postherpetic neuralgia (PHN), may develop at a risk of 10–18% [6]. Typically, PFP is self-limited, but long-term poor outcomes do occur when it is under a severe condition at onset. The use of corticosteroids can help attenuate disease progression by decompressing the swelling caused by inflammation of the facial nerve canal [7]. As for Ramsay Hunt syndromes, the anti-viral drugs such as famciclovir or acyclovir are used to reduce inflammatory reaction from Varicella-Zoster Virus(VZV) [5].

Apart from the choice of medicine, the timing of intervention is critical. An early combined acyclovir-corticosteroid therapy displays a better result exclusive usage of corticosteroid or antiviral therapy [7]. Moreover, over 80% of the patients who receive antiviral therapy within 72 h from onset could obtain better recovery [8]. Otherwise, sequelae such as round eye or synkinesis may appear over time [9, 10]. Thus, the use of oral steroids within 72 h of symptom onset is recommended by modern medicine [11].

Although the onset is regardless of age and gender, a North American study suggests that children have a higher incidence of Bell’s palsy [12]. In the setting of no treatment, 70% of the Bell’s palsy patients are fully recovered, 15% progress to non-flaccid PFP and 15% recover with minor deficits. For people who are unable to restore by themselves, a potentially devastating disorder may result in functional, sensuous, and psychosocial sequelae on patients [13]. Corticosteroids have adverse effects on the brain’s developing or metabolic system, osteoporosis and adrenal insufficiency could occur after a long time of use [14, 15]. The use of glucocorticoids or antiviral drugs is slightly restricted until the age of 18 [16]. An alternative therapy like acupuncture is needed. There is no age limitation, and many clinical studies have found that the intervention of early acupuncture and moxibustion programs can shorten the course of PFP, reduce complications, and the sequelae [17–20].

Acupuncture is defined as inserting fine needles into specific locations in the body [21]. When treating PFP, the timing of acupuncture intervention is also important. A large number of clinical experiences suggest that the earlier patients get treated by acupuncture, the better. But for a long time, there are concerns that acupuncture therapy would aggravate facial nerve edema and cause a delay in recovery [11, 22]. This recognition may be due to a lack of knowledge about acupuncture therapy. In fact, acupuncture is not a simple procedure by choosing some local points near the lesion, but a purposeful and targeted operation under the guidance of Traditional Chinese Medicine (TCM) theory.

There is an old saying in Chinese medicine, *When there is sufficient healthy qi inside, pathogenic factors(evil qi)have no way to invade the body*. Thus, illness often indicates that the evil qi has overcome the healthy qi. TCM believes that the main cause of facial paralysis is the emptiness of the meridian and the invasion of external evils. In more detail, evil wind plays the most important part no matter in Bell’s palsy or Ramsay Hunt syndrome. Combined with emotional factors, dampness-heat of liver and gallbladder or hyperactivity of liver yang are common syndrome differentiation types of Ramsay Hunt syndrome [23].

Modern studies indicate that acupuncture therapy has a certain effect in the treatment of autoimmune diseases and neurological diseases, this provides a theoretical support for people using acupuncture to treat facial neuritis. Studies also show that acupuncture can regulate unbalanced emotions [24–26]. As mentioned above, bad mood could be the trigger of PFP, facial paralysis may result in functional or psychosocial sequelae such as depression or anxiety on the contrary [13, 27]. Therefore, the regulation of emotions by acupuncture provides a role in the treatment.

Acupuncture can balance the body condition and strengthen healthy energy by tonifying qi, activating blood, dredging collaterals by acupoints’ stimulating. A proper selection of acupoints should consider the location of lesion and the syndrome differentiations. On top of that, the combination of near part (of lesion) and the distant part (of lesion) could reinforce each other mutually. This is the “horizontal dimension”.

Just as a location is determined by latitude and longitude in a map, the exact location of the needling position should be determined from both horizontal and vertical dimension. When evil qi invaded the body, it usually transmits from the surface layer to the deeper layers inside the body. This process is refined as “pi” “mai” “rou” “jin”, and “gu” by TCM, which could roughly correspond to “skin” “vessel” “flesh’ “tendons” and “bones” anatomically speaking. The depth of the evil qi determines the depth of the insertion layer of acupoints.

During the acute phase of facial paralysis, evil qi is about in the “pi” or “mai” level, and the depth of needle insertion should be relatively shallow. From the perspective of modern medical theory, facial nerve edema still progressing at acute stage, deep needling and strong stimulation could aggravate facial disorder or induce side effects. Therefore, the depth of needle insertion must not be ignored throughout the entire treatment. This is the “vertical dimension”.

**Fig. 1:**
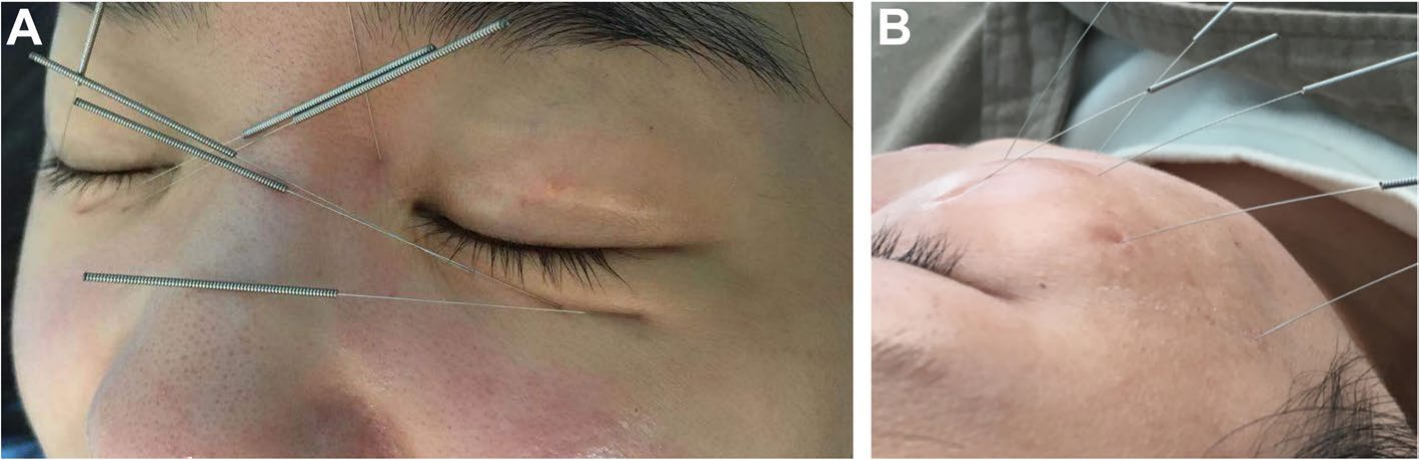
**A**, **B** Shallow and superfacial. When manipulating the shallow needling method, the needle just penetrates through the skin, and the insertion depth is 0.1–0.2 cm

**Fig. 2:**
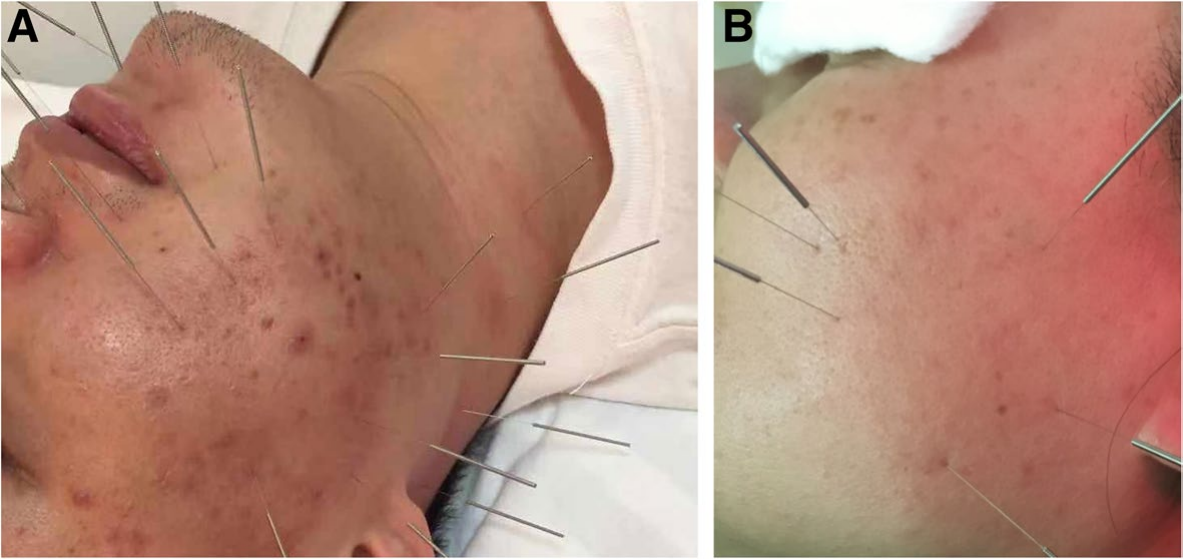
**A**, **B** Deep and penetrated. When manipulating the deep or penetrated method, the insertion may through different tissue levels, or from one acupoint to another acupoint. Insertion depth depending on the lesion level or the level that we aim to achieve

By combining all these factors, the selection of acupoints and acupuncture manipulation gives more precision and effectiveness. When it is on the acute stage of facial paralysis, localized acupoints with superficial manipulation (Fig. 1A, B) is appropriate. While on the recovery stage, local acupoints and penetrating needling should be applied (Fig. 2A, B).

After sorting out the process of acupuncture, concerns about the side effects of early intervention may dispelled. Many clinical studies have found that the intervention of early acupuncture and moxibustion programs can shorten the course of PFP, reduce complications, and the sequelae [17–20]. Whether acupuncture therapy should be involved in the treatment of PFP patients at onset is of great significance.

Four systematic reviews have assessed the safety and effectiveness of acupuncture applying at onset of PFP [28–31], the findings show a beneficial effect. However, methodological limitations from study design or comparisons still exits. Two out of four systematic reviews searched literature only from Chinese databases, the choice of Jadad scale could compromised the quality of their study [29, 30]. We also notice that one of the studies [31] is stricter by limit the control group’s acupoints. All of the systematic reviews focused on the Bell’s palsy without Ramsay Hunt syndrome，and these studies only pay attention to general descriptions such as safety and effectiveness of acupuncture therapy. Specify the most suitable point selection or manipulation at onset of PFP is ignored. Thus, although several systematic reviews are suggesting that the acupuncture treatment at onset is beneficial to the recovery of facial paralysis, there is still controversy about acupuncture therapy.

Recently, more clinical trials have been published which give us more potential evidence about whether there is a most suitable way to perform acupuncture in the acute phase.

## Review objectives

The objective of this systematic review is to assess whether acupuncture should be used in the acute phase of peripheral facial paralysis and whether an early acupuncture intervention can shorten the recovery time of the facial function and reduce the formation of sequelae, and then find out the most suitable therapy for PFP in the acute phase.

## Method

The protocol of this review follows the Preferred Reporting Items for Systematic Review and Meta-Analysis Protocols checklist (online Supplemental file 1) [32], and was registered at PROSPERO international register of systematic reviews (No. CRD42020205127). The method used for this protocol will be performed under the criteria of the Cochrane Handbook of Systematic Reviews of Interventions [33].

### Criteria of studies

There is no restriction on publication language or types. When there are studies reported in different languages other than Chinese or English, we will ask professionals for help.

#### Type of studies

We will include all RCTs and quasi-RCTs of acupuncture for peripheral facial palsy. If there is a cross-over trial, we will only use the first phase data and outcomes for analyzing.

#### Type of participants

According to TCM theory, the causes of PFP such as Bell’s palsy or Ramsay Hunt syndromes are inseparable from “wind-evil”, so there are same features in treatment. All participants who are in the acute phase of PFP will be included regardless of gender, age, or race.

#### Type of intervention

Acupuncture therapy including manual acupuncture, electroacupuncture, moxibustion, bloodletting, and fire needle around acupoints will be eligible, there will be no limitation on acupuncture points, stimulation techniques, stimulation methods, the depth of needle insertion, needle retention time, stimulation frequency, and so on. If there is a co-therapy with acupuncture, it is necessary to be consistent with the control group.

#### Type of control

Blank control, placebo, or treatment such as antiviral therapy, nutritional nerve therapy will be eligible. If both groups involve acupuncture, it is acceptable for the control group not to undergo acupuncture in the acute phase. Or if the control group applies a different acupuncture method from no matter techniques or depth, will all be eligible. To avoid interference from other TCM medication, the combination of Herbal treatment will be ruled out.

#### Type of outcome

When facial symmetry can be restored and how to reduce the sequelae of facial palsy are the most common concerns of outpatients. Thus, the primary outcomes will be the recovery rate of facial function, the time it takes to restore facial function and the odds of sequelae occurring (use House-Brackman scales, Facial Nerve Grading 2.0 and other related scales). Chronic facial discomfort can cause anxiety or depression, and emotional factors are integral to the treatment in Chinese medicine, our secondary outcomes will be psychological well-being and quality of life (use Facial Disability Index scale (FDI), World Health Organization Quality of Life Scale-Brief Form Questionnaire(WHOQOL-BREF)).

### Patient and public involvement

No patient involved in the design, conduct parts of the research. Four of our outpatients permitted us to use their photos during acupuncture treatment as a more intuitive explanation of different kinds of acupuncture manipulation.

### Search strategy

Eight major English and Chinese electronic databases will be searched: PubMed, Embase, Cochrane Library, Web of Science, CNKI (China National Knowledge Infrastructure), VIP (China Science Technology Journal Database), Wanfang Database, Sino-Med Database (including China Biology Medicine disc (CBM)). Search dates: from their inception to December 31th, 2022. LC will apply several strategy exercises in different electronic databases to adjust the sensitivity and specificity (online Supplemental file 2, the detailed search strategy in PubMed). Unpublished literatures such as conference report, dissertation, ongoing studies that meet the eligibility criteria will also be searched. CADTH Grey matters cheklist (www.cadth.ca/resources/finding-evidence/grey-matters), Chinese Clinical Trials Registry (www.chictr.org.cn) and Us National Institutes of Health Ongoing Trials Register (www.clinicaltrials.gov) will be searched to reduce potential publication bias.

### Selection of studies

**Fig. 3:**
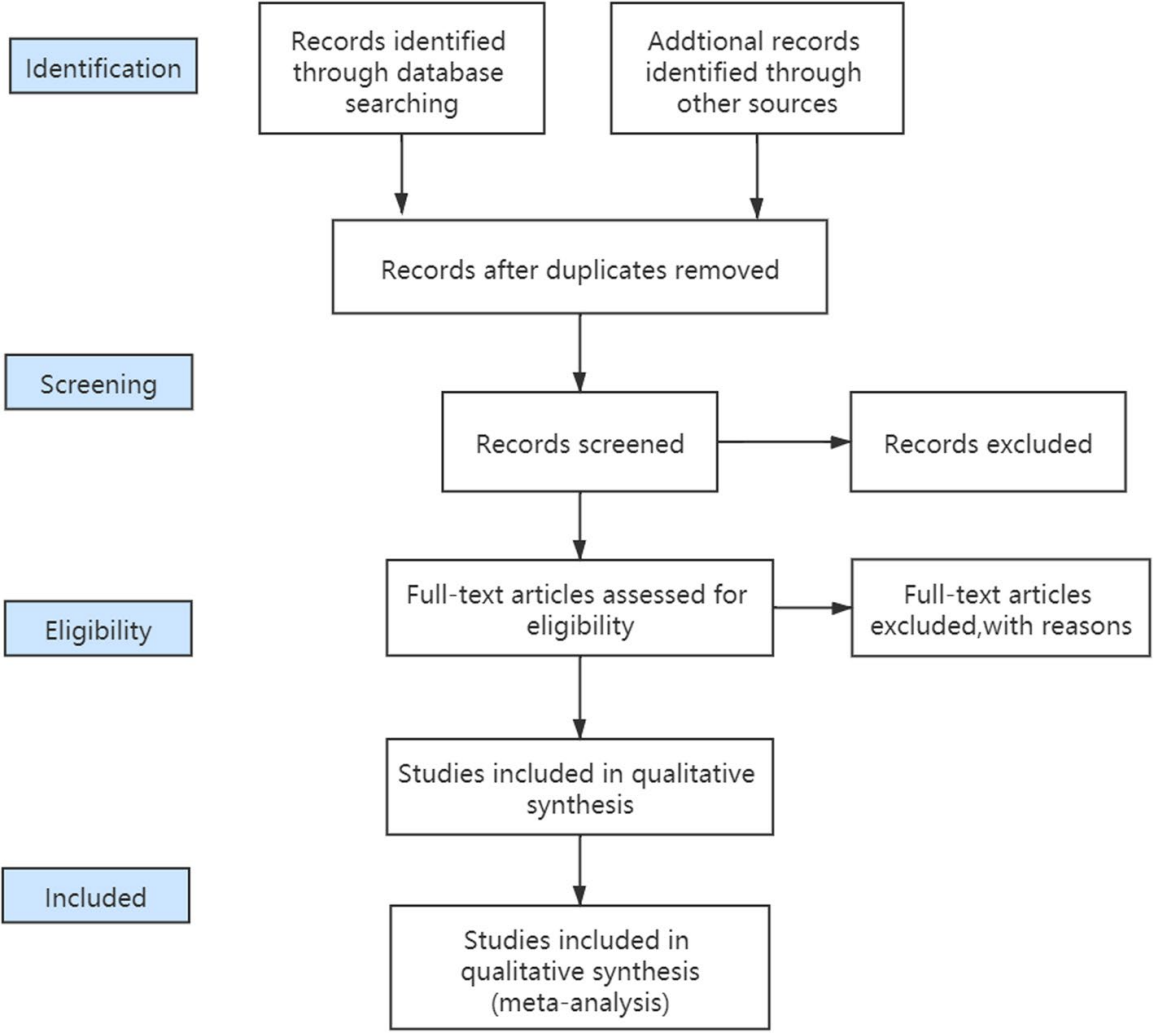
Flow chart of literature selection

We will choose software such as EndNote and NoteExpress to manage studies searching from different databases. Two reviewers (XLL and YY) will respectively screen the title and abstracts according to the predefined criteria. Then full-text phases of the review will also be done by these two authors. If there are any disagreements, a third reviewer will join in and resolve them after discussion (Fig. 3).

### Data extraction

Two reviewers (XLL and SQM) will extract the data independently from the included RCTs and quasi-RCTs by using EXCEL. The following information will be included: (1) Basic information of research: authors, year, title, diseases, corresponding author, contact details, reviewer’s ID, time of extraction. (2) Study designs: type of study, sample size, method of random number generation and allocation concealment, blinding, incomplete outcome data, selective reporting. (3) Patient characteristics: inclusion and exclusion criteria, age, gender, diagnostic criteria, severity, race, institution, baseline comparability. (4) Intervention group: type/form of acupuncture therapy, acupuncture points, manipulation, frequency, course of treatment. (5) Control group: control treatment, operation, frequency, course of treatment. (6) Outcomes: effectiveness outcome, safety outcome, efficiency. The discrepancies will be resolved by a third reviewer through consensus.

### Quality assessment

Two reviewers (SHD and SQM) will evaluate the quality of the selected studies independently according to the version 2 of the Cochrane risk-of-bias tool (RoB 2) for randomized control trials. The following domains of bias will be assessed: bias arising from the randomization process, bias due to deviations from intended interventions, bias due to missing outcome data, bias in measurement of the outcome, bias in selection of the reported result. By response signalling questions, and then give the risk of bias judgement [34].

### Data synthesis and analysis

If studies are judged to be homogeneous, we will perform a meta-analysis. We will pool the data using risk ratio or odds ratio with 95% confidence interval (CI) for dichotomous outcomes, as for continuous outcomes, we will use standard mean difference or mean difference with 95% CI. The clinical heterogeneity will be assessed according to the characteristics of the included studies and participants, details of the intervention or control, types of outcome measurements will also be analyzed. The *I*^2^ statistic will be used to assess the heterogeneity. According to the Cochrane handbook, when *I*^2^ is between 0 and 40%, 30 and 60%, 50 and 90%, and 75 and 100%, the heterogeneity will be regarded as not important, moderate, substantial, and considerable. We will use the random-effects model to conduct the meta-analysis unless the *I*^2^ statistic is 75%. When there is substantial heterogeneity, we will try to investigate possible causes from clinical perspectives. Subgroup and sensitivity analysis will be conducted. Forest plots will show the results of the meta-analysis, and if there are more than 10 included studies, funnel plots willed be used to identify publication bias.

### Subgroup analysis

For facial paralysis, there are different severity and TCM syndrome; for intervention, there are different types of acupuncture therapy; and there are different types of the control group. These all can lead to heterogeneity. To explore the treatment effects respectively, we plan to conduct subgroup analysis for different severity, syndromes, acupuncture techniques (such as manuacupuncture, electroacupuncture, a combination of a different method, different stimulate of acupoint, different acupoint selection, different depth of needling), control group, and other possible factors.

### Sensitivity analysis

For the main outcome with important positive significance, when the literature conditions are met, the random method is compared according to the methodological quality of the literature. Clear/unclear, double-blind use or not; when the combined results are in a critical state and the heterogeneity is small, compare results of random effects model and fixed effects model. If necessary, leave each study out to assess the weight of the research.

### Quality of the evidence

Summary of Findings (SoF) table will be generated by the GRADEpro Guideline Development Tool (GDT). The SoF tables will show the overall quality of the body of evidence. Whether to upgrade or downgrade the level of the evidence will according to the Grading of Recommendations, Assessment, Development, and Evaluation (GRADE) criteria [35]. Study quality limitations, inconsistency of effect, imprecision, indirectness and potential publication bias will downgrade the quality of the evidence.

## Discussion

Although acupuncture therapy has been widely spread over the world by its curative effect on many diseases, how to choose the most suitable treatment for different conditions may not been recognized by the public or even practitioners. When facing facial paralysis, the sooner the treatment the better reach a consensus between western medicine and TCM. In order to maximize the value of acupuncture treatment, we're now trying to gather the evidence and analyze it. Current difficulties are the quality of the original literature and the complex treatments of acupuncture.

Until now, there have been clinical studies on acupuncture treatment which showed effectiveness and safety. A few systematic reviews have concluded that acupuncture is effective at onset of PFP. However, previous studies ignored the specific acupuncture therapies and manipulation. That’s one of the reasons why clinical conclusions were not detailed enough to be a reference for clinical practitioners.

In this study, different PFP in the acute phase will all be pooled. There were no restrictions on acupuncture therapy, and no restrictions on the specific treatment of the control group. With there is more research about applying treatment in its acute phase of PFP, we can review as many different acupuncture therapies as possible. By pooling all these studies, the heterogeneity will be increased, which seems to be a defect. But through appropriate subgroup analysis, we may explore the source of heterogeneity and evaluate the influence of grouping factors on the results. Due to the particularity of acupuncture therapy, the practitioner cannot perform blinding, so we plan to slightly relax the standard for the quality evaluation of this part. The blinding of outcome assessment will be conducted.

## Supplementary Information

**Additional file 1.** PRISMA-P (Preferred Reporting Items for Systematic review and Meta-Analysis Protocols) 2015 checklist: recommended items to address in a systematic review protocol*

**Additional file 2.** Searching strategy on PubMed
