# Supplementary material for: Should acupuncture therapy be used for acute facial paralysis? A protocol for systematic review
Source: Syst Rev. 2023 Mar 15;12:43. doi: 10.1186/s13643-023-02194-5 (PMC10015935; doi:10.1186/s13643-023-02194-5)
Supplement: Supplementary file 2 — Additional file 2. Searching strategy on PubMed [file 13643_2023_2194_MOESM2_ESM.pdf]

Searching strategy on Pubmed

#1 Facial paralysis[Title/Abstract])

#2 Paralysis, Facial[Title/Abstract]

#3 Paralyses, facial[Title/Abstract]

#4 Facial Palsy[Title/Abstract]

#5 Facial Palsies[Title/Abstract]

#6 Palsy, Facial[Title/Abstract]

#7 Palsies, Facial[Title/Abstract]

#8 Hemifacial Paralysis[Title/Abstract]

#9 Paralysis, Hemifacial[Title/Abstract]

#10 Paralyses, Hemifacial[Title/Abstract]

#11 Facial Paresis[Title/Abstract]

#12 Paresis, Facial[Title/Abstract]

#13 Pareses, Facial[Title/Abstract]

#14 Facial Palsy, Lower Motor Neuron[Title/Abstract]

#15 Lower Motor Neuron Facial Palsy[Title/Abstract]

#16 Facial Paralysis, Peripheral[Title/Abstract]

#17 Facial Paralyses, Peripheral[Title/Abstract]

#18 Paralysis, Peripheral Facial[Title/Abstract]

#19 Peripheral Facial Paralysis[Title/Abstract]

#20 Bell palsy[Title/Abstract]

#21 Bell Palsies[Title/Abstract]

#22 Palsy, Bell[Title/Abstract]

#23 Palsies, Bell[Title/Abstract]

#24 Facial Neuropathy, Inflammatory, Acute[Title/Abstract]

#25 Facial Paralysis, Idiopathic[Title/Abstract]

#26 Facial Paralyses, Idiopathic[Title/Abstract]

#27 Idiopathic Facial Paralysis[Title/Abstract]

#28 Idiopathic Facial Paralyses[Title/Abstract]

#29 Paralysis, Idiopathic Facial[Title/Abstract]

#30 Paralyses, Idiopathic Facial[Title/Abstract]

#31 Inflammatory Facial Neuropathy, Acute[Title/Abstract]

#32 Acute Inflammatory Facial Neuropathy[Title/Abstract]

#33 Facial Neuropathy, Idiopathic Acute[Title/Abstract]

#34 Idiopathic Acute Facial Neuropathy[Title/Abstract]

#35 Bell's Palsy[Title/Abstract]

#36 Bell's Palsies[Title/Abstract]

#37 Bells Palsy[Title/Abstract]

#38 Palsies, Bell's[Title/Abstract]

#39 Palsy, Bell's[Title/Abstract]

#40 Acute Idiopathic Facial Neuropathy[Title/Abstract]

#41 Herpetic Facial Paralysis[Title/Abstract]

#42 Facial Paralysis, Herpetic[Title/Abstract]

#43 Facial Paralyses, Herpetic[Title/Abstract]
#44 Herpetic Facial Paralyses[Title/Abstract]
#45 Paralysis, Herpetic Facial[Title/Abstract]
#46 Paralyses, Herpetic Facial[Title/Abstract]
#47 Hunt's syndrome[Title/Abstract]
#48 Ramsay Hunt Syndrome[Title/Abstract]
#49 or/1-48
#50 acute[Title/Abstract]
#51 phase, acute[Title/Abstract]
#52 stage, acute[Title/Abstract]
#53 period, early[Title/Abstract]
#54 acute phase[Title/Abstract]
#55 acute stage[Title/Abstract]
#56 early period[Title/Abstract]
#57 or/50-56
#58 acupuncture treatment[Title/Abstract]
#59 acupuncture therapy[Title/Abstract]
#60 therapy, acupuncture[Title/Abstract]
#61 electroacup*[Title/Abstract]
#62 point[Title/Abstract]
#63 acup*[Title/Abstract]
#64 treatment, acupuncture[Title/Abstract]
#65 fire needle[Title/Abstract]
#66 needling[Title/Abstract]
#67 skin needle[Title/Abstract]
#68 moxibustion[Title/Abstract]
#69 moxabustion[Title/Abstract]
#70 bloodletting therapy[Title/Abstract]
#71 or/58-70
#72 randomly[Title/Abstract]
#73 trial[Title/Abstract]
#74 groups[Title/Abstract]
#75 randomized controlled trail[Title/Abstract]
#76 controlled clinical trail[Title/Abstract]
#77 randomi?ed[Title/Abstract]
#78 placebo[Title/Abstract]
#79 drug therapy[Title/Abstract]
#80 or/72-79
#81 49 and 57
#82 81 and 71
#83 82 and 80
